# Impact of Multiple Viral Detections by FilmArray® on the Severity of Pediatric Respiratory Infections: A Retrospective Single-Center Study

**DOI:** 10.7759/cureus.95864

**Published:** 2025-10-31

**Authors:** Shinya Tomori, Kazuhiro Takahashi, Masakazu Mimaki

**Affiliations:** 1 Pediatrics, Teikyo University School of Medicine, Tokyo, JPN

**Keywords:** children, co-infection, hmpv, multiplex pcr, respiratory tract infections, rhinovirus, rsv, sars-cov-2, severity

## Abstract

Introduction

FilmArray® Respiratory Panel (FA) is a rapid multiplex polymerase chain reaction (PCR) assay that can detect various viruses. While some studies have indicated that viral co-infections might exacerbate severity, others have found no notable difference when compared to single-virus infections, leaving the overall impact uncertain.

Methods

We retrospectively reviewed pediatric patients diagnosed with viral respiratory infections who underwent FA testing at the Teikyo University Hospital between April 2020 and March 2025. Only cases of viral detection were included. FA detects pathogens, including severe acute respiratory syndrome coronavirus 2 (SARS-CoV-2), human metapneumovirus (hMPV), human rhinovirus/enterovirus, influenza A and B viruses, human parainfluenza virus type 1-4, respiratory syncytial virus (RSV), common coronaviruses, and adenovirus. Clinical severity indicators were compared between the single- and multiple-virus groups using multivariate regression. Patients with *Bordetella pertussis*, *Bordetella parapertussis*, *Chlamydophila pneumoniae*, and *Mycoplasma pneumoniae* detected by FA were excluded because bacterial infection was the primary cause. Although secondary bacterial pneumonia often follows viral infection, cases with significant bacteria identified through sputum or blood cultures were excluded as bacterial pneumonia cases. Moreover, patients with a positive rapid test for group A β-hemolytic streptococcus and suspected streptococcal infection were excluded from the analysis.

Results

Of 122 patients (median age 1.8 years), 83 (68.0%) had single-virus detection and 39 (32.0%) had multiple-virus detection. Rhinovirus/enterovirus (38.2%), RSV (17.6%), and hMPV (14.7%) were the most common viruses detected. There were no significant differences in hospital stay (median 6.0 days, p=0.43) between the groups. Similarly, there were no significant differences in clinical severity, chest retraction (OR=2.00, p=0.10, 95% CI=0.87-4.82), pneumonia (OR=1.06, p=0.88, 95% CI=0.48-2.36), poor oral intake (OR=0.76, p=0.48, 95% CI=0.34-1.65), oxygen requirement (OR=0.96, p=0.92, 95% CI=0.44-2.14), tachypnea (OR=0.81, p=0.62, 95% CI=0.36-1.86), or worsening symptoms after admission (OR=1.18, p=0.77, 95% CI=0.39-4.06) between the groups. In addition, subgroup analyses were conducted based on age group, presence or absence of underlying medical conditions, presence or absence of RSV co-infection, co-infections with rhinovirus/enterovirus and single-virus infection, and co-infections with rhinovirus/enterovirus and those without rhinovirus/enterovirus. However, no significant differences were observed between groups.

Conclusion

Viral detection counts from multiplex PCR should not solely guide treatment, as clinical findings remain crucial for assessing severity. While multiple detections did not correlate with worse outcomes, the limited sample size requires careful interpretation. Further prospective studies are needed to determine the significance of viral codetection in children.

## Introduction

The FilmArray® Respiratory Panel (FA), a multiplex polymerase chain reaction (PCR) assay for respiratory infections, has been reimbursed by Japan’s National Health Insurance System since 2019. Capable of detecting 17 viruses and three bacteria within approximately 60 min, FA exhibits high sensitivity (84.4% to 100%) and specificity (89.1% to 100%) [1]. Its use has been linked to reductions in antibiotic use and length of hospital stay [2]. Although the ability of FA to identify multiple pathogens is a key advantage, the clinical implications of codetection in respiratory tract infections remain uncertain. To date, no study has examined whether detecting two or more viruses using FA is associated with clinical severity of respiratory infections in children, including physical examination findings.

The present study assessed the impact of multiple viral detection using FA on the severity of pediatric respiratory infections at our institution.

## Materials and methods

Study population

We retrospectively reviewed the medical records of pediatric outpatients and inpatients who underwent FA testing at the Teikyo University Hospital between April 2020 and March 2025. All patients who underwent FA presented with clinical symptoms during their hospital visit. Eligible patients were those diagnosed with respiratory tract infections, including acute upper respiratory infection, bronchiolitis, bronchitis, or pneumonia, who tested positive exclusively for viral pathogens by FA. In the context of FA, *Bordetella pertussis*, *Bordetella parapertussis*,* Chlamydophila pneumoniae*, and *Mycoplasma pneumoniae *may also be identified. However, patients in whom these pathogens were detected were excluded from the study population because bacterial infection was deemed the primary cause. In addition, cases in which the rapid test for group A β-hemolytic *streptococcus* was positive and a group A β-hemolytic streptococcal infection was clinically suspected were determined to be primarily due to bacterial infection and were excluded from the analysis. Additionally, although secondary bacterial pneumonia following a viral infection is common, distinguishing between the two is challenging, and antibiotics are sometimes administered to treat bacterial pneumonia in such instances [3]. Nevertheless, cases in which significant bacteria were identified through sputum or blood culture were excluded from the study population as definitive cases of bacterial pneumonia.

Measurement methods

Nasopharyngeal swab specimens from eligible patients were analyzed using FilmArray® Respiratory Panel v2.1 (bioMérieux, Marcy-l'Etoile, France). This assay detects adenovirus, common coronaviruses (OC43, HKU1, NL63, and 229E), severe acute respiratory syndrome coronavirus 2 (SARS-CoV-2), human metapneumovirus (hMPV), human rhinovirus/human enterovirus, influenza A virus, influenza B virus, human parainfluenza virus type 1-4, and respiratory syncytial virus (RSV) [4]. As FA does not differentiate between rhinoviruses and enteroviruses, it is considered to be a single pathogen.

Statistical analyses

All statistical analyses were performed using the JMP Pro version 18.2.1 (SAS Institute Inc., Cary, NC, USA). In the primary analysis, multivariate linear and logistic regression analyses were used to evaluate the association between the number of viruses detected and the disease severity. The primary outcome was length of hospital stay. Secondary outcomes included severity classification of community-acquired pneumonia and previously reported clinical severity indicators, such as pneumonia on chest radiographs, poor oral intake/dehydration, chest retractions, apnea, oxygen requirement, tachypnea, impaired consciousness, circulatory failure, number of positive severity indicators, and clinical deterioration after admission (e.g., new oxygen requirement, initiation of antibiotics, and onset of tachypnea or retractions) [5,6]. Tachypnea was defined as >60 breaths/min in neonates, >50 breaths/min in infants, >40 breaths/min in toddlers, and >20 breaths/min in school-aged children [5]. Age and presence of underlying conditions were included as covariates. Median values with interquartile ranges (IQRs) have been reported. To mitigate the risk of a type I error arising from multiple testing of secondary outcomes, a Bonferroni correction was implemented. The adjusted significance threshold was established at p<0.0083, calculated by dividing 0.05 by the six tests conducted. Odds ratios (ORs) with 95% confidence intervals (CIs) and p-values are reported. A p-value <0.05 was deemed statistically significant for the primary outcome. Patients were classified as having underlying conditions if they presented with neurological, respiratory, or chromosomal disorders; congenital heart disease; or a history of preterm birth or very low birth weight [7-9]. Subsequently, subgroup analyses were conducted by age group (infant, toddler, preschool children, school-age children, and adolescents), underlying conditions, RSV co-detection, and rhinovirus/enterovirus co-detection. Consistent with the primary analysis, we evaluated the length of the hospital stay and clinical indicators. The Wilcoxon rank-sum test was used to assess the length of hospital stay, and Fisher’s exact test was used as a clinical indicator. To examine patient characteristics, the Wilcoxon rank-sum test was applied to continuous variables, whereas Fisher’s exact test was used for categorical variables, comparing multiple infections with a single infection. In subgroup and patient characteristic analyses, a p<0.05 was deemed statistically significant.

Ethical approval

This study was approved by the Ethics Review Board for Medical and Health Research at Teikyo University (Approval No. 25-025). All procedures involving human participants adhered to the ethical standards of the institutional and/or national research committees, the 1964 Declaration of Helsinki and its subsequent amendments, and the equivalent ethical standards.

## Results

**Figure 1:**
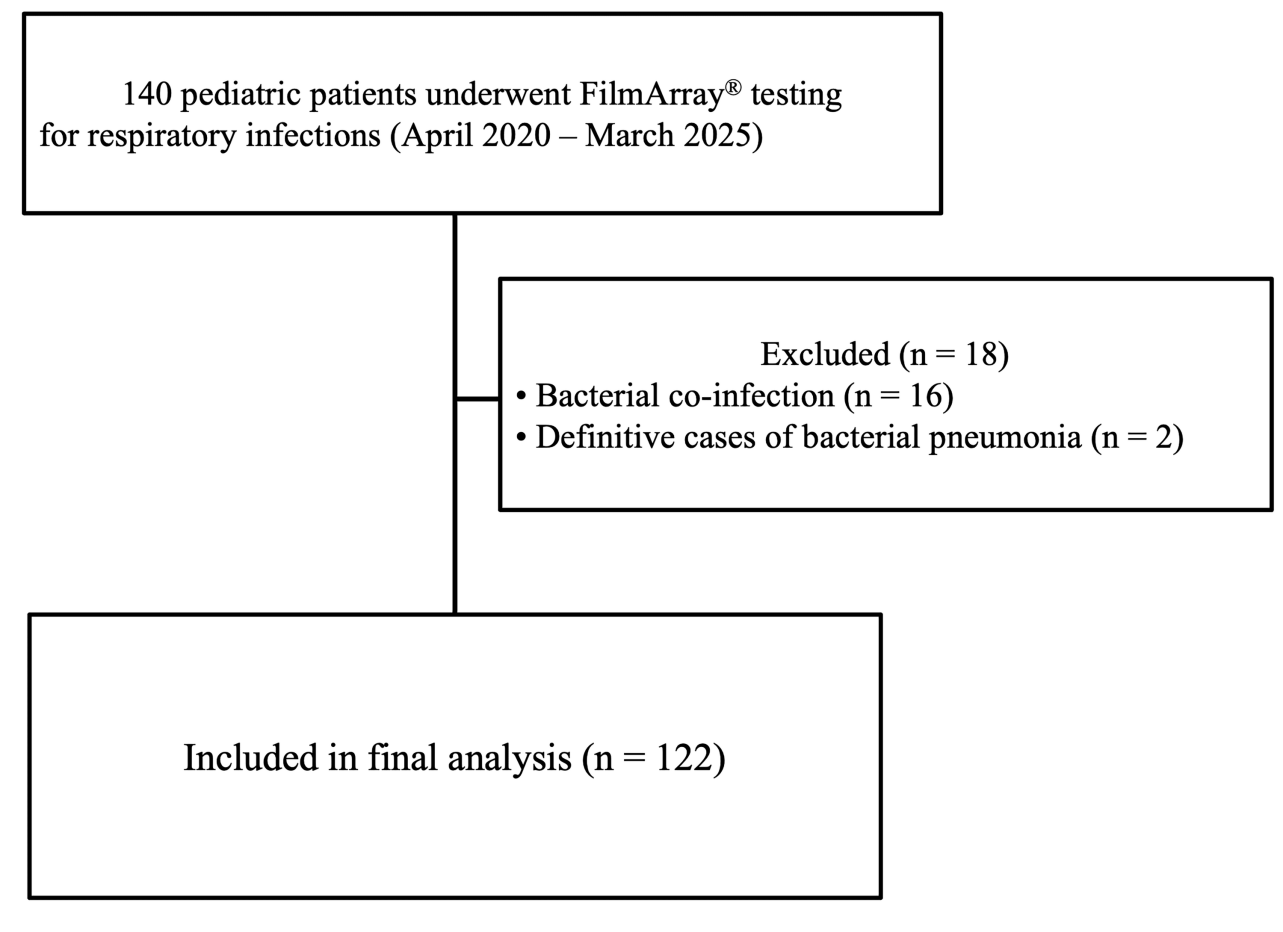
Patient flow diagram of the study cohort

Of the 140 pediatric patients who underwent FA, 18 were excluded because of bacterial co-infection (n=16) or confirmed cases of bacterial pneumonia (n=2). Consequently, 122 patients were included in the final analysis (Figure 1).

The patient characteristics are presented in Table 1.

**Table 1:** Patient characteristics IQR: interquartile range

Parameters (n=122)	n (%) or median (IQR)
Median age (IQR)	1.8 (1.0-4.0)
Male sex, n (%)	86 (70.5%)
Underlying disease, n (%)	71 (58.1%)
- Neurological disorders	20 (28.2% of 71)
- Respiratory diseases such as bronchial asthma or chronic lung disease	19 (26.8% of 71)
- A history of preterm birth or very low birth weight	15 (21.1% of 71)
- Chromosomal abnormalities	12 (17.0% of 71)
- Immunodeficiency	3 (4.2% of 71)
- Congenital heart disease	2 (2.8% of 71)
Number of detected viruses	1 virus: 83 (68.0%)
	2 viruses: 29 (23.8%)
	3 viruses: 9 (7.4%)
	4 viruses: 1 (0.8%)

None of the patients presented with impaired consciousness, apnea, or circulatory failure. Therefore, these variables were excluded from the analysis. The median age at the time of testing was 1.8 (IQR: 1.0-4.0) years old. Of the total cohort, 86 patients (70.5%) were male, and 71 (58.1%) had underlying conditions. The most frequent underlying disease categories included neurological disorders (n = 20, 28.2%), respiratory diseases such as bronchial asthma or chronic lung disease (n = 19, 26.8%), history of preterm birth or very low birth weight (n = 15, 21.1%), chromosomal abnormalities (n = 12, 17.0%), immunodeficiency (n = 3, 4.2%), and congenital heart disease (n = 2, 2.8%). FA identified one virus in 83 patients (68.0%), two viruses in 29 (23.8%), three viruses in nine (7.4%), and four viruses in one (0.8%). In addition, we compared the characteristics of patients with multiple infections to those of patients with a single infection, but no significant differences were observed (Table 2).

**Table 2:** Comparison of patient characteristics between multiple and single infections IQR: interquartile range

Parameters (n=122)	Single virus (n=83)	Multiple viruses (n=39)	P-value
Median age (IQR)	2.0 (1.0-5.0)	1.6 (1.1-2.0)	0.44
Male sex, n (%)	58 (69.9%)	28 (71.8%)	0.83
Underlying disease, n (%)	50 (60.2%)	20(51.3%)	0.43

The detected viruses are listed in Table 3, and 170 were identified.

**Table 3:** Viruses detected by FA RSV; respiratory syncytial virus; hMPV: human metapneumovirus; SARS-CoV-2: severe acute respiratory syndrome coronavirus 2; FA: FilmArray® Respiratory Panel

Detected virus (n=170)	Number of positive	Proportion
Rhinovirus/enterovirus	65	38.2%
RSV	30	17.6%
hMPV	25	14.7%
Parainfluenza virus type 3	17	10.0%
Adenovirus	13	7.6%
Influenza A	4	2.4%
Parainfluenza virus type 4	4	2.4%
SARS-CoV-2	3	1.8%
Coronavirus HKU1	3	1.8%
Parainfluenza virus type 1	2	1.2%
Coronavirus NL63	2	1.2%
Coronavirus OC43	1	0.6%
Influenza B virus	1	0.6%

Rhinovirus/enterovirus was the most frequently detected virus (n=65, 38.2%) and the most commonly co-detected virus (n=28, 33.3%) (Table 4).

**Table 4:** Viruses in co-infections RSV; respiratory syncytial virus; hMPV: human metapneumovirus; SARS-CoV-2: severe acute respiratory syndrome coronavirus 2

Detected virus in co-infections (n=84)	Number of positive	Proportion
Rhinovirus/enterovirus	28	33.3%
RSV	14	16.7%
Adenovirus	11	13.1%
hMPV	9	10.7%
Parainfluenza virus type 3	9	10.7%
Parainfluenza virus type 4	4	4.7%
SARS-CoV-2	3	3.6%
Coronavirus HKU1	3	3.6%
Coronavirus NL63	2	2.4%
Coronavirus OC43	1	1.2%

Primary analysis

The median hospital stay was six (IQR: 4.0-8.0) days in the single-virus group and six (IQR: 4.0-7.0) days in the multi-virus group (p=0.43). No significant differences were observed between the groups in terms of clinical severity indicators, including chest retraction (OR=2.00, p=0.10), pneumonia on imaging (OR=1.06, p=0.88), poor oral intake/dehydration (OR=0.76, p=0.48), oxygen requirement (OR=0.96, p=0.92), tachypnea (OR=0.81, p=0.62), or worsening of symptoms after admission (OR=1.18, p=0.77) (Table 5).

**Table 5:** Comparison of clinical severity between single-virus and multiple-virus infections OR: odds ratio; CI: confidence interval

Clinical indicator	Total cases (n=122)	Single virus (n=83)	Multiple viruses (n=39)	OR (95% CI)	P-value
Chest retractions	46 (37.7%)	35 (42.2%)	11 (28.2%)	2.00 (0.87-4.82)	0.10
Pneumonia on imaging	58 (47.5%)	39 (47.0%)	19 (48.7%)	1.06 (0.48-2.36)	0.88
Poor oral intake/dehydration	58 (47.5%)	37 (44.6%)	21 (53.9%)	0.76 (0.34-1.65)	0.48
Oxygen requirement	51 (41.8%)	35 (42.2%)	16 (41.0%)	0.96 (0.44-2.14)	0.92
Tachypnea	45 (36.9%)	30 (36.1%)	15 (38.4%)	0.81 (0.36-1.86)	0.62
Worsening symptoms after admission	19 (17.0%)	14 (18.2%)	5 (14.3%)	1.18 (0.39-4.06)	0.77

The residual analysis revealed no marked deviation from normality or homoscedasticity.

Subgroup analysis

Infants (<1 Year)

Among the 26 infants, 20 had single-virus infections and six had multiple-virus infections. The median hospital stay was six (IQR: 4.0-7.0) days in the single-virus group and 4.5 (IQR: 3.5-9.3) days in the multi-virus group (p=0.76). No significant differences were observed between the groups. Chest retractions were more common in the single-virus group (OR=0.16, p=0.17), while pneumonia was not observed in any multiple-virus cases. No significant differences were observed for any of the other clinical indicators (Table 6).

**Table 6:** Comparison of clinical severity between single-virus and multiple-virus infections in infants OR: odds ratio; CI: confidence interval

Clinical indicator	Total cases (n=26)	Single virus (n=20)	Multiple viruses (n=6)	OR (95% CI)	P-value
Chest retractions	12 (46.2%)	11 (55.0%)	1 (16.7%)	0.16 (0.02-1.67)	0.17
Pneumonia on imaging	8 (30.8%)	8 (40.0%)	0	N/A	0.13
Poor oral intake/dehydration	18 (69.2%)	14 (70.0%)	4 (66.7%)	0.86 (0.12-6.01)	1.00
Oxygen requirement	7 (26.9%)	5 (25.0%)	2 (33.3%)	1.50 (0.21-10.82)	1.00
Tachypnea	9 (34.6%)	7 (35.0%)	2 (33.3%)	0.92 (0.13-6.40)	1.00
Worsening symptoms after admission	4 (15.4%)	3 (15.0%)	1 (16.7%)	1.06 (0.09-12.68)	0.96

Toddlers and Preschool Children (1-6 Years)

No significant association was identified in this group (n=74). For example, chest retractions (OR=0.79, p=0.80) and oxygen requirements (OR=1.17, p=0.81) were comparable (Table 7). Regarding the median hospital stay, there was no significant difference: six (IQR: 5.0-8.0) days in the single-virus group and six (IQR: 5.0-7.0) days in the multi-virus group (p=0.76).

**Table 7:** Comparison of clinical severity between single-virus and multiple-virus infections in toddlers and preschool children OR: odds ratio; CI: confidence interval

Clinical indicator	Total cases (n=74)	Single virus (n=45)	Multiple viruses (n=29)	OR (95% CI)	P-value
Chest retractions	28 (37.8%)	18 (40.0%)	10 (34.5%)	0.79 (0.30-2.08)	0.80
Pneumonia on imaging	43 (58.1%)	25 (55.6%)	18 (62.1%)	1.31 (0.50-3.40)	0.64
Poor oral intake/dehydration	33 (44.6%)	15 (51.7%)	15 (54.5%)	1.60 (0.63-3.48)	0.35
Oxygen requirement	34 (45.6%)	20 (44.4%)	14 (48.2%)	1.17(0.46-2.97)	0.81
Tachypnea	27 (36.5%)	11 (37.9%)	16 (35.6%)	1.17 (0.42-2.91)	1.00
Worsening symptoms after admission	11 (15.9%)	4 (14.8%)	7 (16.7%)	0.87 (0.23-3.31)	1.00

School-Aged Children and Adolescents (≥7 Years)

Among the 22 cases, including only four multiple infections, statistical significance was not observed for any indicator. Pneumonia (OR=0.67, p=1.00) and tachypnea (OR=1.57, p=1.00) were not significantly different. Although the OR for poor oral intake/dehydration was high, no significant difference was observed (OR=2.6, p=0.56) (Table 8). The median length of hospital stay was six (IQR: 4.0-11.0) days in the single-virus group and 2.5 (IQR: 2.0-3.0) days in the multiple-virus group. Although no significant difference was observed, there was a tendency for longer hospital stay in the single-virus group (p=0.05).

**Table 8:** Comparison of clinical severity between single-virus and multiple-virus infections in school-aged children and adolescents OR: odds ratio; CI: confidence interval

Clinical indicator	Total cases (n=22)	Single virus (n=18)	Multiple viruses (n=4)	OR (95% CI)	P-value
Chest retractions	6 (27.3%)	6 (33.3%)	0	N/A	0.54
Pneumonia on imaging	7 (31.3%)	6 (33.3%)	1 (25.0%)	0.67 (0.06-7.85)	1.00
Poor oral intake/dehydration	7 (31.8%)	5 (27.8%)	2 (50.0%)	2.6 (0.28-23.8)	0.56
Oxygen requirement	10 (45.5%)	10 (55.6%)	0	N/A	0.10
Tachypnea	9 (43.8%)	7 (38.9%)	2 (50.0%)	1.57 (0.18-13.9)	1.00
Worsening symptoms after admission	4 (18.2%)	4 (22.2%)	0	N/A	1.00

Underlying conditions

Among the 71 patients, 51 had single-virus infections, and 20 had multiple-virus infections. There were no significant differences in any clinical severity indicators between the two groups. For instance, chest retractions were observed in 47.1% of the single-virus group and in 35.0% of the multiple-virus group (OR=0.61, p=0.43). Similarly, the proportions of patients with pneumonia on imaging (OR=1.64, p=0.42), oxygen requirement (OR=0.69, p=0.60), and tachypnea (OR=1.31, p=0.60) were not significantly different (Table 9). In terms of the median duration of hospital stay, no notable difference was observed: six days (IQR: 4.0-9.0) for the single-virus group compared to 4.5 days (IQR: 4.0-8.0) for the multi-virus group (p=0.63).

**Table 9:** Comparison of clinical severity between single-virus and multiple-virus infections in children with underlying conditions OR: odds ratio; CI: confidence interval

Clinical indicator	Total cases (n=71)	Single virus (n=51)	Multiple viruses (n=20)	OR (95% CI)	P-value
Chest retractions	31 (43.7%)	24 (47.1%)	7 (35.0%)	0.61 (0.21-1.77)	0.43
Pneumonia on imaging	26 (36.9%)	17 (33.9%)	9 (45.0%)	1.64 (0.57-4.70)	0.42
Poor oral intake/dehydration	33 (46.5%)	23 (45.1%)	10 (50.0%)	1.21 (0.43-3.43)	0.79
Oxygen requirement	33 (46.5%)	25 (49.0%)	8 (40.0%)	0.69 (0.24-1.98)	0.60
Tachypnea	32 (43.8%)	22 (41.5%)	10 (50.0%)	1.31 (0.47-3.72)	0.60
Worsening symptoms after admission	15 (22.7%)	11 (23.4%)	4 (21.1%)	0.87 (0.24-3.18)	1.00

Respiratory syncytial virus co-detection

Among the 39 co-infected patients, 13 had RSV co-infection and 26 had co-infections without RSV. No significant differences were found in the frequency of pneumonia on imaging (OR=0.86, p=1.00) or oxygen requirement (OR=0.85, p=1.00). Although tachypnea was more frequent in the RSV co-infection group, the difference was not statistically significant (OR=2.62, p=0.19). In contrast, although there was no significant difference, there was a tendency for chest retractions to be more common in cases of single infection (OR=0.68, p=0.71) (Table 10). Regarding the median length of hospital stay, there was no significant difference noted: five days (IQR: 4.5-8.0) for the group with RSV co-infection compared to six days (IQR: 4.0-6.3) for those with multiple viral infections excluding RSV (p=0.78).

**Table 10:** Comparison of clinical severity between RSV co-infection and multiple viral infections other than RSV OR: odds ratio; CI: confidence interval; RSV: respiratory syncytial virus

Clinical indicator	Total cases (n=39)	RSV co-infection (n=13)	Multiple infections other than RSV (n=26)	OR (95% CI)	P-value
Chest retractions	11 (28.2%)	3 (23.1%)	8 (30.8%)	0.68 (0.14-3.13)	0.71
Pneumonia on imaging	19 (48.7%)	6 (46.2%)	13 (50.0%)	0.86 (0.26-3.26)	1.00
Poor oral intake/dehydration	21 (53.8%)	7 (53.8%)	14 (53.8%)	1.00 (0.26-3.80)	1.00
Oxygen requirement	16 (41.0%)	5 (38.5%)	11 (42.3%)	0.85 (0.22-3.32)	1.00
Tachypnea	15 (38.5%)	7 (53.8%)	8 (30.8%)	2.62 (0.67-10.35)	0.19
Worsening symptoms after admission	9 (23.1%)	2 (15.4%)	7 (26.9%)	1.15 (0.17-7.99)	1.00

Co-infections without rhinovirus/enterovirus and single-virus infection

When comparing co-infections without rhinovirus/enterovirus (n=11) to single-virus infections (n=83), no statistically significant differences were found in chest retractions (OR=0.30, p=0.19), pneumonia (OR=3.01, p=0.20), or oxygen requirement (OR=1.14, p=1.00) (Table 11). When comparing the median hospital stay lengths, no significant difference was found: patients with a single viral infection had a median stay of five days (IQR: 4.0-8.0), while those with co-infections, excluding rhinovirus/enterovirus, also had a median stay of five days (IQR: 3.8-6.0) (p=0.12).

**Table 11:** Comparison of clinical severity between single viral infection and co-infections without rhinovirus/enterovirus OR: odds ratio; CI: confidence interval

Clinical indicator	Total cases (n=94)	Single virus (n=83)	Co-infections without rhinovirus/enterovirus (n=11)	OR (95% CI)	P-value
Chest retractions	37 (38.9%)	35 (41.6%)	2 (18.2%)	0.30 (0.06-1.50)	0.19
Pneumonia on imaging	47 (50%)	39 (47.0%)	8 (72.7%)	3.01 (0.75-12.1)	0.20
Poor oral intake/dehydration	42 (44.7%)	37 (44.6%)	5 (45.4%)	1.04 (0.29-3.66)	1.00
Oxygen requirement	40 (42.6%)	35 (42.2%)	5 (45.4%)	1.14 (0.32-4.04)	1.00
Tachypnea	35 (37.2%)	30 (36.1%)	5 (45.4%)	1.47 (0.41-5.23)	0.74
Worsening symptoms after admission	15 (17.1%)	14 (18.0%)	1 (10.0%)	0.51 (0.06-4.34)	0.52

Co-infections with rhinovirus/enterovirus and those without rhinovirus/enterovirus

Among the 39 co-infected patients, 28 had co-infections, including rhinovirus/enterovirus, while 11 did not. Clinical indicators, such as chest retraction (OR=2.13, p=0.47) and pneumonia (OR=0.24, p=0.08), did not differ significantly. Although pneumonia tended to be more frequent in non-rhinovirus co-infections, the difference was not statistically significant. Conversely, worsening symptoms after admission and chest retractions appeared to be more prevalent in instances of rhinovirus/enterovirus co-infection (Table 12). The median length of hospital stay was six days (IQR: 5.0-8.0) in co-infections with rhinovirus/enterovirus and five days (IQR: 3.8-6.0) in co-infections without rhinovirus/enterovirus, with no significant difference observed (p=0.14).

**Table 12:** Comparison of clinical severity between co-infections with rhinovirus/enterovirus and those without rhinovirus/enterovirus OR: odds ratio; CI: confidence interval

Clinical indicator	Total cases (n=39)	Co-infections with rhinovirus/enterovirus (n=28)	Co-infections without rhinovirus/enterovirus (n=11)	OR (95% CI)	P-value
Chest retractions	11 (28.21%)	9 (32.1%)	2 (18.2%)	2.13 (0.38-12.0)	0.47
Pneumonia on imaging	19 (48.8%)	11 (39.3%)	8 (72.7%)	0.24 (0.05-1.12)	0.08
Poor oral intake/dehydration	21 (53.9%)	16 (57.1%)	5 (45.5%)	1.60 (0.39-6.51)	0.73
Oxygen requirement	16 (41.0%)	11 (39.3%)	5 (45.5%)	0.78 (0.19-3.18)	0.74
Tachypnea	15 (38.5%)	10 (35.7%)	5 (45.5%)	0.67 (0.16-2.75)	0.72
Worsening symptoms after admission	5 (14.3%)	4 (16.0%)	1 (10.0%)	1.71 (0.17-17.6)	1.00

## Discussion

This study demonstrated no significant differences in clinical severity indicators between children with multiple viruses detected by FA and those with single-virus infections. Although the OR for chest retractions was relatively elevated, no significant differences were observed for other indicators or respiratory deterioration following admission, suggesting that multiple viral detections by FA are not associated with increased severity of pediatric respiratory infections. Additionally, subgroup analyses did not show any consistent associations with clinical severity, implying that there might be a weak link between the number of viruses detected and the severity of respiratory infections in children. Although some studies have compared the clinical impact of single versus multiple viral infections, none have specifically addressed this association by using FA [6,10,11].

Cilla et al. reported higher hospitalization rates among children with community-acquired pneumonia and multiple viral infections than those with single-virus infections [12]. Studies on acute bronchiolitis have similarly shown that approximately one-third of hospitalized patients present with co-infections, which may be linked to greater severity or longer hospital stays [10,11,13]. Notably, Richard et al. conducted a study in infants hospitalized for bronchiolitis and found that dual viral infections were associated with a significantly higher risk of pediatric intensive care unit (PICU) admission, although the length of hospital stay did not differ significantly [13]. In the context of viral co-infection, particularly with human rhinovirus and RSV, two hypotheses have been proposed regarding increased disease severity. One study suggested that a diminished interferon-γ response associated with RSV may facilitate human rhinovirus replication. Another study proposed that RSV-infected airway epithelial cells upregulate the expression of intercellular adhesion molecule-1 (ICAM-1) on the cell surface, thereby creating a foundation for more severe human rhinovirus infection through ICAM-1, the principal receptor for human rhinovirus [11].

In contrast, Martin et al. reported that children with single-virus infections had a higher risk of oxygen requirement, prolonged hospitalization, and ICU admission [6]. Additionally, up to 27% of asymptomatic children under the age of six years have been shown to carry at least one respiratory virus [14,15]. Rhinovirus and seasonal coronaviruses, frequently identified even in asymptomatic individuals, were among the most commonly co-detected viruses in this study, suggesting that some viral detection may represent asymptomatic carriage rather than active infection [14]. Nevertheless, viruses infrequently detected in asymptomatic individuals, such as RSV and hMPV, were also frequently observed in co-infections, indicating that the presence of multiple viruses may not directly influence disease severity [14]. Similarly, a systematic review and meta-analysis by Asner et al., which included 21 studies and 4280 patients, found no significant differences in length of stay, oxygen requirement, or mortality between children with viral co-infections and those with single-virus infections, except in a subgroup of preschool-aged children where mortality was elevated [16]. Similarly, Goka et al. reported in their systematic review that mixed viral infections were present in 5%-62% of pediatric cases (mean approximately 23%), although the association with clinical severity varied, with several studies indicating no clear link between co-infection and severe outcomes [17]. These findings align with our results, suggesting that the detection of multiple respiratory viruses alone does not inherently indicate increased clinical severity and that host-related factors and pathogen-specific virulence likely play more influential roles. One possible explanation is that the host immune response to an initial infection may attenuate the impact of subsequent infections through interferon-mediated and other antiviral mechanisms [6]. For example, infection with HRV or other RNA viruses triggers an antiviral interferon response in infected human airway cells through recognition of viral nucleic acids by innate immune sensors. This response induces the production of type I and III interferons and activates antiviral ISGs. Consequently, replication of subsequently invading viruses in the airway is thought to be suppressed [18].

In addition to its diagnostic benefits, appropriate interpretation of multiplex PCR results is essential for antimicrobial stewardship. Overdiagnosis due to incidental viral detection may lead to unnecessary antibiotic prescriptions, particularly in pediatric patients with overlapping viral and bacterial symptoms. Integrating FA results with comprehensive clinical evaluations can reduce diagnostic uncertainty and prevent the overuse of antibiotics in viral respiratory infections [1].

However, several limitations of FA testing must be acknowledged. As a qualitative assay, FA does not provide quantitative viral load data nor can it differentiate between active infection and prolonged viral shedding. Asymptomatic carriage, particularly rhinoviruses and seasonal coronaviruses, can lead to detection that is not clinically relevant. Furthermore, the timing of testing relative to symptom onset may affect the probability of detecting multiple viral genomes; early sampling may capture transient or residual co-detections without a clinical impact. Therefore, the FA results should be interpreted in a broader clinical and epidemiological context.

Moreover, clinical judgment is crucial when evaluating FA results. Management decisions should not be based solely on the number of viruses detected, as clinical presentation and patient history are vital in this context. Consequently, FA findings should be considered within a broader clinical scenario, especially for very young infants (<3 months), immunocompromised children, and those with pre-existing cardiopulmonary conditions, who may need more vigilant monitoring even if multiple co-detections appear clinically harmless.

Several limitations of the present study warrant mention. First, this study was conducted retrospectively at a single center, which may constrain the generalizability of the findings. As the utilization of FA has become more prevalent across diverse hospital settings, the results of this study may not comprehensively represent broader clinical practices or populations. Moreover, with a total of 122 patients, the study may have lacked sufficient power to detect statistically significant differences in several outcomes despite the presence of potential clinical trends (e.g., OR 2.00; chest retractions, p=0.10). Additionally, our multivariate models were limited to adjustments for age and underlying conditions, omitting several potentially significant confounding variables, such as seasonality, prior antibiotic use, vaccination status, and the interval between symptom onset and sample collection. These unmeasured factors may have influenced both the probability of virus detection and the severity of clinical outcomes. To enhance the external validity and applicability of these findings, multicenter studies with larger sample sizes encompassing varied geographic regions and institutional practices are recommended. Second, the FA is a qualitative assay that does not quantify viral load. For example, previous quantitative PCR studies have indicated that rhinovirus loads exceeding 10^4.5^ copies/mL are strongly associated with symptomatic infections [14]. But FA provides only qualitative results, lacking quantitative viral load indicators such as cycle threshold (Ct) values or viral copy numbers. This limitation hinders our ability to distinguish clinically significant viral replication from incidental or residual viral genome detection, particularly in cases of multiple viral detections. Additionally, the timing of testing during the course of illness (e.g., early vs. late phase) may influence the probability of detecting viral nucleic acids, potentially contributing to variability in detection patterns. These factors should be considered when interpreting the significance of co-detected viruses, and future studies incorporating quantitative PCR metrics and standardized sampling timing are warranted. Third, the severity evaluations were based on a medical record review, which may be subject to observer bias and incomplete documentation. Fourth, the study period encompassed five years (April 2020 to March 2025), thereby ensuring that data collection covered all seasons. Consequently, potential seasonal variations in viral respiratory infections were included in our cohort, with no specific seasons (e.g., winter months) being excluded. However, it is important to note that the analysis was not adjusted for seasonality, which may have affected the distribution of specific viruses and the observed clinical severity. Finally, because this study included only patients who underwent FA testing, there is a potential for selection bias. FA testing is generally performed in hospitalized patients. As a result, our cohort may have been disproportionately composed of children with more complex or severe clinical courses, which could limit the generalizability of our findings to all pediatric respiratory infection cases.

## Conclusions

This study suggests that the number of viruses detected using multiplex PCR should not guide treatment decisions. Clinical findings remain paramount in determining disease severity. Although multiple detections were not significantly associated with worse outcomes, the limited sample size and potential asymptomatic viral shedding warrant cautious interpretation. Larger prospective studies are needed to clarify the clinical relevance of viral co-detection in children.
